# Radiofrequency Echographic Multi Spectrometry (REMS) Technology for Bone Health Status Evaluation in Kidney Transplant Recipients

**DOI:** 10.3390/diagnostics14182106

**Published:** 2024-09-23

**Authors:** Angelo Fassio, Giovanni Adami, Stefano Andreola, Pietro Manuel Ferraro, Paola Pisani, Fiorella Anna Lombardi, Ombretta Viapiana, Maurizio Rossini, Chiara Caletti, Giovanni Gambaro, Matteo Gatti, Davide Gatti

**Affiliations:** 1 Rheumatology Unit, University of Verona, 37134 Verona, Italy; adami.g@yahoo.com (G.A.); ombretta.viapiana@univr.it (O.V.); maurizio.rossini@libero.it (M.R.); matgat93@gmail.com (M.G.); davide.gatti@univr.it (D.G.); 2 Nephrology Unit, University of Verona, 37134 Verona, Italy; stefano.andreola@aovr.veneto.it (S.A.); pietromanuel.ferraro@univr.it (P.M.F.); chiara.caletti@aovr.veneto.it (C.C.); giovanni.gambaro@hotmail.it (G.G.); 3 Institute of Clinical Physiology, National Research Council, 37100 Lecce, Italy; pisanipaolaifc@gmail.com (P.P.); dott.ssafalombardi@gmail.com (F.A.L.)

**Keywords:** REMS, bone mineral density, kidney transplant recipients, osteoporosis diagnosis, bone health status

## Abstract

**Background**: A significant loss in bone density and strength occurs during the post-renal-transplant period with higher susceptibility to fracture. The study aims to compare the performance of the Radiofrequency Echographic Multi Spectrometry (REMS) in the bone mineral density assessment with the conventional dual-energy X-ray absorptiometry (DXA) in a cohort of kidney transplant recipients (KTR). **Methods**: A cohort of 40 patients underwent both DXA and REMS examinations on the lumbar spine and/or proximal femur. The paired *t*-test was used to compare DXA and REMS measurements; the chi-square test was used to compare the prevalence of osteoporosis/osteopenia. The agreement between the two techniques was assessed through Spearman’s correlation. **Results**: As expected, most KTR patients were osteopenic or osteoporotic with both REMS and DXA (86.5% and 81% for the femur; 88% and 65% for the lumbar spine *p* < 0.05). A modest correlation (r = 0.4, *p* < 0.01) was observed at the lumbar spine between the T-score measured by REMS and DXA. A strong correlation was defined between REMS and DXA in the femoral region (r = 0.7, *p* < 0.0001). **Conclusions**: The study demonstrates the exchangeability of the two techniques on the proximal femur in KTR and a higher diagnostic accuracy of REMS at the spine level than DXA.

## 1. Introduction

Chronic Kidney Disease (CKD) is tightly associated with alterations of the mineral bone metabolism coupled with the occurrence of osteoporosis and fragility fractures [1]. Among CKD patients, kidney transplant recipients (KTR) are at high risk of premature mortality compared with the general population as a result of significant skeleton and cardiovascular-related disorders [2,3]. Bone fractures are a common and severe clinical condition in these patients compared to the general population [4,5,6]. Indeed, after a kidney transplant, the most common abnormalities of bone metabolism involve hypophosphatemia, vitamin D deficiency, hypocalcemia, and hyperparathyroidism that overall affect bone loss, consequently increasing the risk of fracture [5]. With respect to the complications encountered by CKD and KTR patients, the National Institutes of Health (NIH) defined osteoporosis as “a skeletal disorder characterized by compromised bone strength predisposing to an increased risk of fracture. Bone strength reflects the integration of two main features—bone density and bone quality” [7]. This definition suggests the relevance of monitoring bone strength and fracture risk for the management of CKD. Both variables are orchestrated by several factors, including bone density, microarchitecture, bone turnover, cumulative damage, and osteogenic repair [4]. Therefore, a proper estimation of both bone quantity and quality is of utmost importance in clinical practice. Bone mineral density is the standard parameter for bone quantity that correlates with the content of bone mineral per square centimeter (g/cm^2^) of bone tissue, and it is commonly measured by dual-energy X-ray absorptiometry (DXA). Instead, bone quality refers to bone tissue material composition, which can be evaluated more invasively by using bone biopsy with quantitative histomorphometry [8]. Prior to transplantation, KTR already displayed a significant loss in bone strength, characterized by high rates of osteopenia and osteoporosis, which are reported to be 32% and 15%, respectively [8,9,10], in addition to the occurrence of skeletal fractures [11,12]. A dramatic decrease in bone density up to 9% usually occurs between 12 and 18 months after transplantation [8,13,14]. Although DXA remains the gold standard for the measurement of bone mineral density (BMD), artefacts such as aortic calcifications, which are common in this special population [15], may significantly affect the BMD estimation, especially at the lumbar spine [16]. Radiofrequency Echographic Multi Spectrometry (REMS) is an alternative non-ionizing technique for the BMD assessment of axial reference sites. This technology relies on the automatic processing of unfiltered native ultrasound signals acquired during an echographic scan, which are modulated only by the physical properties of the bone. Therefore, after discarding signals that correspond to artefacts (e.g., aortic calcifications, osteophytes, and prosthesis) [17,18,19], the bone status assessment results from the comparison of the spectral profile of the patient to previously acquired reference models for healthy, or osteoporotic conditions [20,21]. Moreover, REMS has demonstrated optimal sensitivity and specificity in the discrimination of osteoporotic patients, good diagnostic agreement with DXA, and the capacity to provide effective information on bone microarchitecture [22,23,24]. The aim of this cross-sectional exploratory study was to compare REMS with DXA in the BMD assessment of KTR patients and their subsequent classification as osteoporotic, low BMD, or normal BMD.

## 2. Materials and Methods

We enrolled Caucasian men and women who underwent kidney transplantation and were referred to the Nephrology Unit at the University Hospital of Verona between June and December 2020. All subjects fulfilled the following inclusion criteria: age between 40 and 80 years, BMI < 40 kg/m^2^, and a functioning kidney graft. The enrolled patients underwent a DXA examination at the lumbar spine and/or proximal femur, according to their medical prescription, and an echographic scan of the same anatomical sites by REMS, as described below.

BMD values at the lumbar spine and femoral sites were measured for all patients through anteroposterior DXA scans performed according to standard clinical routine procedures using the following device: GE Lunar iDXA 194 system (GE Healthcare Lunar, Madison, WI, USA). Corresponding T- and Z-score values derived from the BMD were obtained from the lumbar spine and femoral neck for the diagnostic classification. In line with World Health Organization (WHO) criteria [25], patients were therefore classified as osteoporotic, having low BMD or within the normal range.

The densitometric data obtained from this device have been preliminarily converted into Hologic equivalent values as described in Di Paola et al. [23]. All the DXA medical reports were anonymized and stored for subsequent analysis.

REMS scans at the lumbar spine and proximal femur were performed with a dedicated echographic device (EchoStation, Echolight Spa, Lecce, Italy) equipped with a convex transducer operating at the nominal frequency of 3.5 MHz and used as recommended by the manufacturer. The echographic scan on the lumbar spine is performed by an ultrasound acquisition of the L1–L4 vertebrae, obtained by placing the convex probe on the patient’s abdomen. For femoral scans, the convex probe is placed parallel to the femoral neck, with the indicator of the probe facing the patient. Once the target bone interface is visualized, the operator is required to set the appropriate scan depth and focus. Subsequently, the software integrated into the REMS device with a proprietary database of reference ultrasound spectral models, as described in Conversano et al. and Casciaro et al. [20,21], is used to calculate REMS-measured BMD values, which are used to derive the corresponding T-score and Z-score values. For the calculation of the BMD, the ultrasound data obtained from the femoral and/or vertebral scans were processed by the algorithm [20,21], which has previously performed a series of spectral and statistical analyses of radiofrequency signals (RF) backscattered by the bone target. Specifically, after the automatic identification of the bone interface and the related region of interest (ROI), the spectral profiles of each patient were classified as “healthy”, “osteopenic”, or “osteoporotic” based on the comparison with reference spectral models stored in the database.

### Statistical Analysis

A Shapiro–Wilk test was performed to assess the normality of the datasets obtained with both techniques. A chi-square test was used to compare proportions, whereas a non-parametric paired Wilcoxon rank test was used to estimate the differences in the BMD, T-scores, and Z-scores between DXA and REMS at the considered anatomical sites. The degree of correlation between DXA- and REMS-measured T-score was quantified by Spearman’s coefficient (r). Moreover, the diagnostic concordance between DXA and REMS for the worst site was also assessed by calculating the percentage of patients being classified in the same diagnostic category (osteoporotic, osteopenic, or healthy) based on the T-scores. A two-tailed *p*-value < 0.05 was considered statistically significant. All analyses were performed with GraphPad Prism v. 8.0.1.

## 3. Results

### 3.1. Study Population

The study enrolled 40 patients; for the lumbar scans, 9 fractured and 30 non-fractured patients; for the femoral scans, 8 fractured and 28 non-fractured patients; for the remaining patients, no details regarding the fracture history are available. Table 1 provides information on the characteristics of those patients whose lumbar spine and femoral neck scans were included in the final analysis.

### 3.2. Diagnostic Classification of DXA and REMS

**Figure 1:**
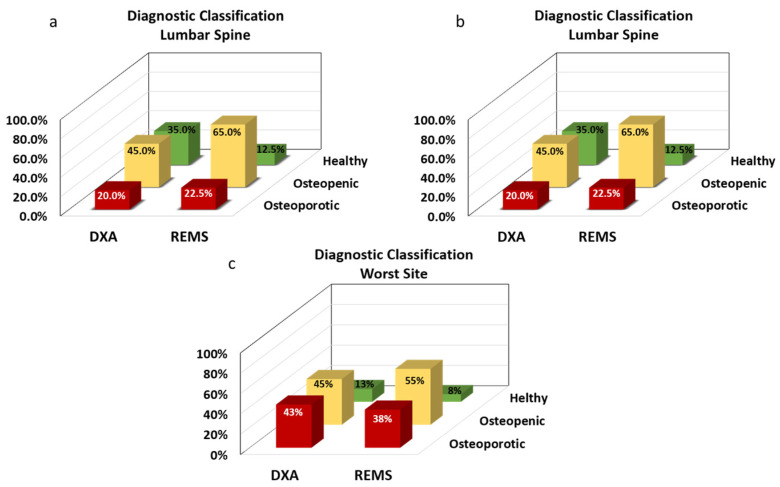
Diagnostic classification into the three categories resulting from a simultaneous DXA and REMS assessment. The proportion of patients diagnosed as osteoporotic, osteopenic, and healthy through DXA and REMS investigations of the lumbar spine (**a**), the femoral neck (**b**), and the worst site (**c**).

Figure 1 depicts the patients’ classification resulting from the DXA and REMS techniques.

REMS acquisitions at the lumbar spine resulted in 88% of patients with osteoporosis or osteopenia diagnosis (23% and 65%, respectively) when compared to the 65% of the DXA technique (20% and 45%, respectively), *p* < 0.05.

Similar results were obtained with both techniques on the femoral neck. The number of patients with osteoporosis or osteopenia was 86.5% (29.7% and 56.8%, respectively) by REMS and 81% (35% and 46%, respectively) by DXA, without significant differences.

When the worst site was considered, the percentage of patients with osteoporosis or osteopenia diagnosis was 93% (38% and 55%, respectively) by REMS and 88% (43% and 45%, respectively) by DXA, without significant differences.

### 3.3. Diagnostic Agreement

**Figure 2:**
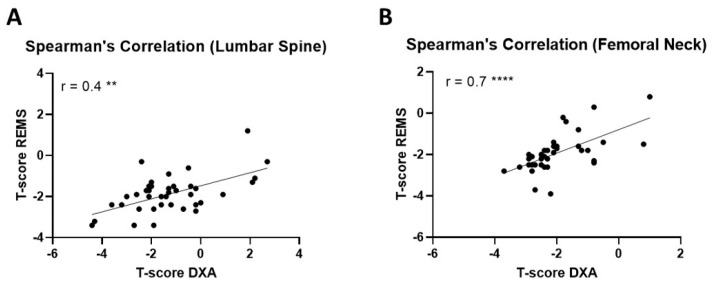
Scatterplot between DXA and REMS measurements at the lumbar spine and femoral neck. The degree of correlation was measured with Spearman’s correlation between DXA and REMS-measured T-scores at the (**A**) lumbar spine and (**B**) femoral neck. ** *p* < 0.01; **** *p* < 0.0001.

As a confirmation of the discrepancy in the diagnostic classification between the two technologies, a modest correlation was found at the lumbar spine between the REMS-measured T-scores and the DXA ones with r = 0.4 (*p* < 0.01) (Figure 2A). In contrast, REMS and DXA showed a strong correlation in the femoral region with r = 0.7 (*p* < 0.0001) (Figure 2B). Comparisons between fractured and non-fractured patients are reported in Tables S1 and S2.

## 4. Discussion

This pilot study explored the results of the BMD assessment obtained with DXA and REMS in a cohort of KTR patients.

In the present sample, we observed significantly higher BMD values at the lumbar spine, as estimated by the DXA, than REMS. This finding might be explained by the presence of unknown fractured vertebrae, aortic calcifications [26], or osteophytes [19], conditions that are known to influence the areal BMD estimated by DXA [15,27]. Recently, in a cohort of patients receiving peritoneal dialysis, we provided further evidence of this phenomenon and observed a promising robustness of the REMS technology in response to these issues [26,28,29]. Contrary to DXA, which is based on a bi-dimensional image projection, REMS provides a BMD measurement through the analysis of ultrasound signals backscattered by the targeted tissues [20]. The functional principle of the ultrasound-based REMS approach involves the analysis of raw, unfiltered ultrasound signals that are preserved on the B-mode image reconstruction, followed by the automatic detection of bone interfaces and related regions of interest (ROI). Unfiltered ultrasound signals derived from each echographic scan line are processed in parallel, and artefacts, such as calcifications, osteophytes, etc., are automatically discarded because of their unexpected spectral profiles in comparison with reference spectral models for pathological or healthy bone [17,18]. A recent report demonstrated the clinical effectiveness of REMS in resolving misinterpretations due to the overestimation of DXA diagnosis on 159 subjects [19]. The study observed that the percentage of women classified as osteoporotic on the basis of the REMS-BMD was considerably higher in comparison to those classified by DXA. The same trend was observed in a subset of patients with osteoarthritis, where the presence of lumbar deformities would produce an untrue BMD increase by DXA densitometry compared to REMS [19,26]. In subjects affected by post-menopausal osteoporosis, longitudinal data showed that REMS is an effective predictor for the risk of fractures, with a significantly higher performance (in terms of area under the curve) of the lumbar REMS-T-scores when compared to the DXA-T-scores [30]. Indeed, REMS has been acknowledged among the novel imaging tools for osteoporosis diagnosis and 5-year prediction of fracture risk assessment [31,32]. Therefore, REMS may represent a valuable strategy for early identification and stratification of high-risk individuals susceptible to fracture following kidney transplantation. As also suggested by KDIGO CKD-MDB 2017 guidelines, for patients with CKD G1T–G5T and with risk factors for osteoporosis, it is recommended to use BMD testing to assess fracture risk.

In the future, prospectively collected data on KTR patients are warranted to monitor the effect of transplantation on bone mass in the long term and to promptly identify patients at risk of fractures by means of REMS.

Our study has its limitations. First, we emphasize that this is an exploratory study with a limited sample size, not sufficient to run a validation process. In addition, there was a lack of any third reference technique, such as quantitative Computed Tomography, to confirm the accuracy of the REMS-derived BMD values.

## 5. Conclusions

This exploratory study investigated, for the first time, the bone health status of KTR recipients, a special population at significant risk of impaired bone health and fragility fractures.

With REMS, most KTR patients fall within the osteopenia/osteoporosis classification, while the DXA measurement might provide a misleading BMD overestimation and consequent spurious increase in the proportion of healthy subjects.

REMS may represent an effective approach for the BMD and fracture risk assessment in the KRT population. The availability of a novel technique for the assessment of BMD, characterised by nimble machinery, absence of ionizing radiations, and good robustness to measurement artefacts, could be extremely useful in everyday clinical practice, also in special populations such as KTR patients.

## Figures and Tables

**Table 1:** Characteristics of the study population. Results are reported as average value ± SD. BMI, body mass index; DXA, dual-energy X-ray absorptiometry; REMS, Radiofrequency Echographic Multi Spectrometry.

	Lumbar Spine	Femoral Neck	Total Hip
Gender	22 men and 18 women	22 men and 18 women	22 men and 18 women
Age (years)	60.43 ± 9.8	58.51 ± 11.2	58.51 ± 11.2
Height (cm)	166.05 ± 9.56	165.54 ± 9.38	165.54 ± 9.38
Weight (kg)	67.1 ± 12.62	65.18 ± 12.67	65.18 ± 12.67
BMI (kg/m^2^)	24.3 ± 4.3	23.8 ± 4.2	23.8 ± 4.2
DXA-BMD	0.929 ± 0.2	0.654 ± 0.1	0.784 ± 0.2
REMS-BMD	0.865 ± 0.1	0.655 ± 0.1	0.795 ± 0.1

## Data Availability

The data that support the findings of this study are available from the corresponding author (A.F.) for privacy.

## Supplementary Materials

The following supporting information can be downloaded at: https://www.mdpi.com/article/10.3390/diagnostics14182106/s1. Table S1: DXA and REMS comparisons between fractured and non-fractured patients. Table S2: Bone health assessment of fractured and non-fractured patients for each reference anatomical site.
