# Supplementary material for: Radiofrequency Echographic Multi Spectrometry (REMS) Technology for Bone Health Status Evaluation in Kidney Transplant Recipients
Source: Diagnostics (Basel). 2024 Sep 23;14(18):2106. doi: 10.3390/diagnostics14182106 (PMC11431431; doi:10.3390/diagnostics14182106)
Supplement: Supplementary file 1 [file diagnostics-14-02106-s001.zip › diagnostics-3203066-supplementary.pdf]

Because of renal impairment, KTR patients experience substantial bone loss which is associated to a reduced T-score and consequent susceptibility to bone fractures. On the basis of the history of fragility fractures collected from each participant, the cohort was stratified into two groups, fractured and non-fractured patients, and the bone health status was evaluated using both techniques. From 40 lumbar scans, 9 and 30 scans encompassed patients with and without a fracture history, respectively; fracture information was not available for 1 patient. Moreover, from 40 femoral examinations, 8 and 28 scans included patients with and without fractures, respectively; fracture information were not available for 4 patients. As expected, the fractured group yielded a lower spinal and femoral T-score values compared to the non-fractured ones with both REMS and DXA techniques. However, only for the scans performed at the lumbar region the measurements differed significantly between the two technologies. In particular, considering the whole cohort of fractured and non-fractured patients, the mean BMD at the lumbar spine obtained with REMS was 0.866 ± 0.104 g/cm2, which was significantly lower in comparison to 0.929 ± 0.191 g/cm2 with DXA ($p<0.05$). Similarly, significant differences were found on the corresponding T- and Z-score values between the two technologies ($p<0.05$). Still at the lumbar region in the non-fractured group, significant differences were found between the REMS-measured BMD, T- and Z-score versus DXA-measured values (all with $p<0.001$). Indeed, in these patients while a T-score of -1.0 ± 1.6 was measured by DXA, the REMS-measured T-score yielded a lower value of -1.8 ± 0.9. Analogously, the Z-score measured at the spine was -0.1 ± 1.3 with DXA and -0.8 ± 0.7 with REMS. The capacity of REMS to detect a more compromised T-score in non-fractured patients was consistent with its tendency, observed previously, to grade a higher fraction of osteopenic/osteoporotic diagnoses compared to those graded by DXA.
On the contrary, REMS- and DXA-based measurements at the femoral neck provided similar results. In the non-fractured group, the average BMD was 0.667 ± 0.143 g/cm2 (T-score = -1.9 ± 1.1) detected by DXA which was comparable to 0.663 ± 0.108 g/cm2 (T-score = -1.9 ± 0.9) measured by REMS. Analogously, in the fractured fraction the measured BMD was 0.607 ± 0.075 g/cm2 (T-score = -2.3 ± 1.4) with DXA and 0.607 ± 0.078 g/cm2 (T-score = -2.3 ± 0.5) with REMS.

Table S1. DXA and REMS comparisons between fractured and non-fractured patients. *$p<0.05$ on the BMD, T-score and Z-score of the total cohort between DXA and REMS; **$p<0.001$ on the BMD, T-score and Z-score of non-fractured patients between DXA and REMS.

**Lumbar Spine**	**n (%)**	**Mean age (± SD)**	***DXA-BMD* (g/cm² ± SD)**	***REMS-BMD* (g/cm² ± SD)**	***DXA T-score* (± SD)**	***REMS T-score* (± SD)**	***DXA Z-score* (± SD)**	***REMS Z-score* (± SD)**
**Fractured**	9 (30%)	63.11 ± 6.7	0.817 ± 0.166	0.819 ± 0.057	-2.3 ± 1.4	-2.3 ± 0.5	-1.1 ± 1.1	-1.2 ± 0.5
**Non-fractured ****	30 (70%)	59.43 ± 10.4	0.963 ± 0.185	0.877 ± 0.110	-1.0 ± 1.6	-1.8 ± 0.9	-0.1 ± 1.3	-0.8 ± 0.7
**Total ***	39 (100%)	60.43 ± 9.8	0.929 ± 0.191	0.866 ± 0.104	-1.3 ± 1.6	-1.9 ± 0.9	-0.3 ± 1.4	-0.8 ± 0.7

Table S2. Bone health assessment of fractured and non-fractured patients for each reference anatomical site. Results are reported as average value ± SD and p-value are obtained using Wilcoxon paired test.

Femoral Neck	n (%)	Mean age (± SD)	*DXA-BMD* (g/cm^2 ± SD)	*REMS-BMD* (g/cm^2 ± SD)	*DXA T-score* (± SD)	*REMS T-score* (± SD)	*DXA Z-score* (± SD)	*REMS Z-score* (± SD)
**Fractured**	8 (29%)	64.38 ± 6.82	0.607 ± 0.075	0.607 ± 0.078	-2.3 ± 0.5	-2.3 ± 0.6	-1.0 ± 0.6	-1.1 ± 0.4
**Non-fractured**	28 (71%)	57.79 ± 10.61	0.667 ± 0.143	0.663 ± 0.108	-1.9 ± 1.1	-1.9 ± 0.9	-0.8 ± 0.9	-0.8 ± 0.6
**Total**	36 (100%)	58.51 ± 11.2	0.654 ± 0.133	0.655 ± 0.108	-2.0 ± 1.0	-1.9 ± 0.9	-0.9 ± 0.9	-0.9 ± 0.6
